# Generation of synthetic microstructures containing casting defects: a machine learning approach

**DOI:** 10.1038/s41598-023-38719-0

**Published:** 2023-07-22

**Authors:** Arjun Kalkur Matpadi Raghavendra, Laurent Lacourt, Lionel Marcin, Vincent Maurel, Henry Proudhon

**Affiliations:** 1 Mines Paris, PSL University, Centre des matériaux (MAT), UMR7633 CNRS, 91003 Evry, France; 2 Safran Aircraft Engines, Etablissement de Villaroche, 77550 Moissy-Cramayel, France

**Keywords:** Engineering, Metals and alloys, Computational methods, Computational science, Statistics

## Abstract

This paper presents a new strategy to generate synthetic samples containing casting defects. Four samples of Inconel 100 containing casting defects such as shrinkages and pores have been characterized using X-ray tomography and are used as reference for this application. Shrinkages are known to be tortuous in shape and more detrimental for the mechanical properties of materials, especially metal fatigue, whereas pores can be of two types: broken shrinkage pores with arbitrary shape and gaseous pores of spherical shape. For the generation of synthetic samples, an integrated module of Spatial Point Pattern (SPP) analysis and deep learning techniques such as Generative Adversarial Networks (GANs) and Convolutional Neural Networks (CNNs) are used. The SPP analysis describes the spatial distributions of casting defects in material space, whereas GANs and CNNs generate a defect of arbitrary morphology very close to real defects. SPP analysis reveals the existence of two different void nucleation mechanisms during metal solidification associated to shrinkages and pores. Our deep learning model successfully generates casting defects with defect size ranging from 100 µm to 1.5 mm and of very realistic shapes. The entire synthetic microstructure generation process respects the global defect statistics of reference samples and the generated samples are validated by statistically comparing with real samples.

## Introduction

Casted materials often carry defects formed during metal solidification. These defects can have a serious impact on the material properties whose magnitude depends on various microstructural and defect characteristics. Some of the defects that can appear in casted materials are shrinkages, pores, oxide films, etc.^1–3^. Shrinkages are large tortuous cavities formed due to contraction of molten metal during solidification whereas pores and micro-voids are smaller in size and are generally formed due to trapped gases. These cavity defects can degrade material performance drastically by promoting initiation and propagation of crack driven by stress concentration^4–7^. The intensity of this degradation depends on various defect characteristics such as its size, position and morphology^8^: the fatigue life is known to vary inversely with respect to defect size, a relationship demonstrated by Kitagawa–Takahashi diagram^9,10^. It is also known that defect location plays a very prominent role in high cycle fatigue (HCF)^10,11^. Cracks initiating from defects that are closer to the free surface propagate faster when compared to those initiating from internal defects given the difference in their stress intensity factors (SIF)^1^. Furthermore, a tortuous morphology of defects can drastically increase stress concentration facilitating crack initiations. Some of the independent features that can characterize defect morphologies are sphericity, aspect ratio, etc.^8^. Whilst these characteristics can induce a large scatter in fatigue life, the problem gets even more complicated in materials containing high porosity levels which results in formation of defect clusters^12^. In clustered defects, apart from the individual features of defects, they are also influenced by the stress gradients of neighbouring defects. These defects can sometimes be found in aeronautical foundry parts like turbine disks and blades, and have received much less attention in mechanical domain. Analysing all the features that might affect fatigue life requires a large number of samples to be tested which can be extremely costly. Therefore, a plausible approach is to generate synthetic microstructures that are very close to reality which can be simulated numerically to create a large database of mechanical response to the presence of defects, their morphology and spatial distribution.

Present work focuses on analysing the effects of defect population in a naturally isotropic material inconel 100 under cyclic loads where the granular characteristics of all tested samples are similar. In such a case, synthetic microstructures can be generated by distributing the defects in a homogeneous material space according to a pattern similar to real defects. The spatial ordering of defects can be analysed through spatial point pattern theory (SPP) with tools like Ripley’s K-function that measures the second order properties of point distribution in space^13^. A similar approach was applied by El Khoukhi et al., where numerical microstructures were generated by placing spherical defects in a homogeneous material space^14^.

### Need for synthetic microstructures

**Figure 1:**
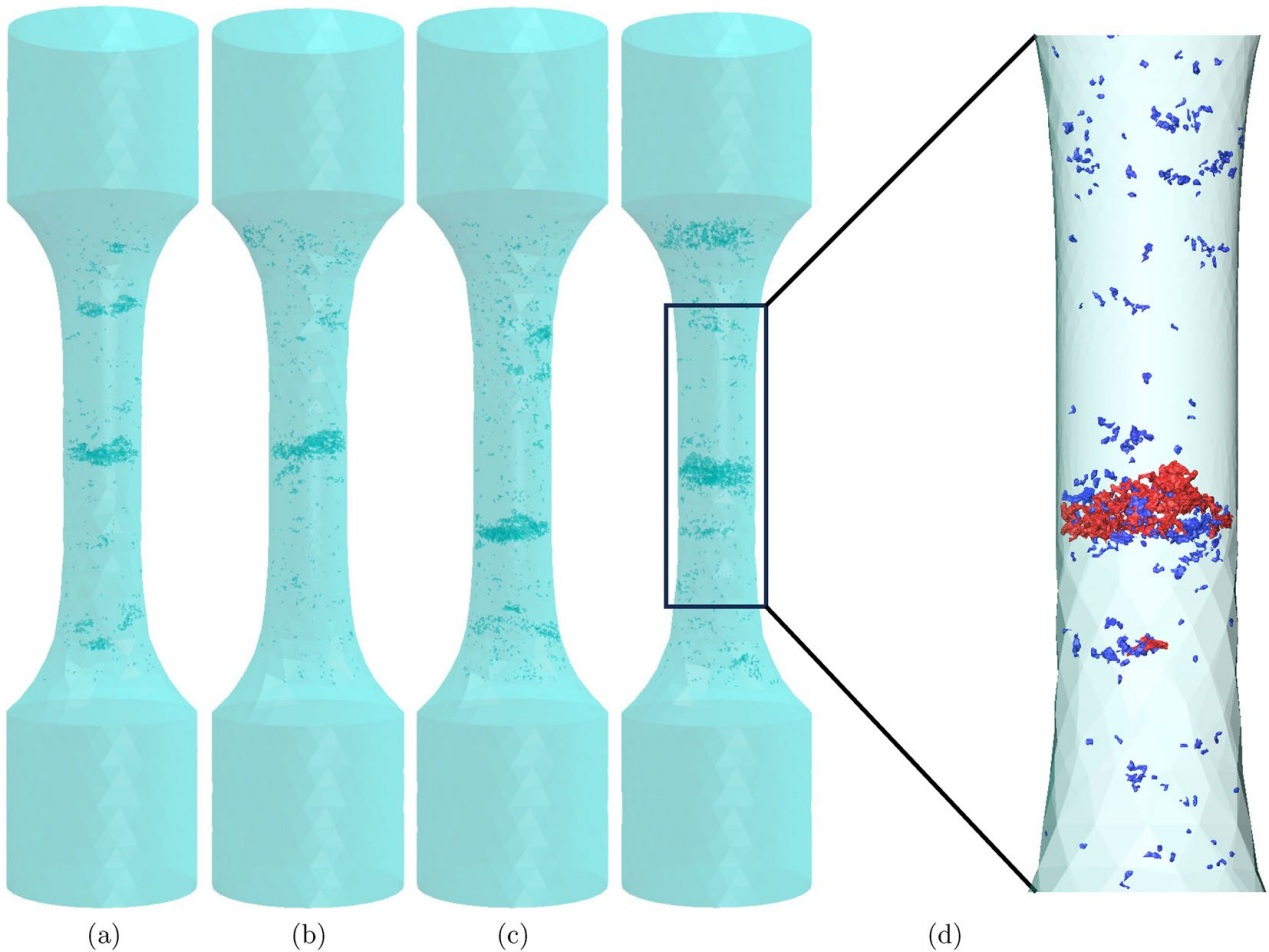
Image-based FE models from X-ray computed tomography (XCT) for reference (**a**) Sample A, (**b**) Sample B, (**c**) Sample C, (**d**) Sample D along with a close up view of the clustered defects in sampled D colored as large defects (red) and small defects (blue). Samples are 40 mm long with gauge diameter of 3.7 mm.

Samples containing clustered defects (see Fig. 1) are known to produce very complex mechanical response under fatigue loading. Although image based finite element (FE) models can simulate this response and aid in locating the crack-initiation site, it is still very difficult to simplify the process and predict its fatigue life with respect to defect characteristics^15^. For an isolated defect, a Kitagawa– Takahashi diagram can be used but the same Linear Elastic Fracture Mechanical (LEFM) approach cannot be applied to clustered defects. Therefore, a better estimation of the parameters influencing the fatigue life apart from just the size of the defect is needed. The additional parameters or features could be the volume fraction of defects, size of the cluster, sphericity, aspect ratio or other morphological parameters. For such an analysis, a large number of samples are needed and generating synthetic microstructures that mimics the real specimens is seemingly an inexpensive approach. Furthermore, these generated microstructures can then be converted to image based FE models and simulated numerically to estimate prominent characteristics or to develop a probabilistic model with an approach similar to Monte-Carlo.

### Reference samples

Four as-cast Inconel 100 (IN100) cylindrical samples machined from cast ingot bars are used to generate synthetic microstructures. The samples are 40 mm long with a gauge diameter of 3.7 mm and contain clustered defects whose characteristics are assessed via X-ray computed tomographic images (XCT) as seen in our previous work^15^. The XCT volumes can be used to build image-based FE models of the same. Via numerical simulations, the critical defect that could initiate primary crack during fatigue loads can be determined. The volumes of the studied XCT scans were 300 mm$^3$.

**Table 1:** Global defect statistics in IN100 samples.

Sample	A	B	C	D
Total no. defects	215	295	346	434
Total defects volume (mm$^3$)	0.935	0.84	1.36	1.58

As seen in Table 1, defects occupy around 0.3–0.52 % of material volume, among which many of these defects are confined in a small thickness along the axis of the sample forming complicated network of defects as seen in Fig. 1. The interaction of these clustered defects is too complicated and requires a profound analysis.

### Method to generate synthetic microstructure

Synthetic microstructures can be generated by placing the defects in a fixed material space of a particular geometry similar to that of real specimens. Placing the defects in material space is a stochastic process which requires a prior understanding of spatial distribution of patterns, for example: via SPP, which is significantly used in the field of astronomy, forestry, cartography etc.^16–20^. With tools like Ripley’s K-function, second order properties of point patterns can be measured: the points in our context are centroids of defect volumes^21^. Neither many researchers have considered SPP analysis to estimate the 3D spatial characterization of defects nor to generate numerical microstructures using the same^14,22^.

In the case of clustered defects, regular shapes cannot be assumed for defects since contributions of various features in degrading material’s performance is merely unknown. Hence, a deep learning strategy called Generative Adversarial Networks (GANs) and Convolutional Neural Networks (CNNs) are integrated together to recreate realistic synthetic defects that can be placed via stochastic process defined by SPP in material space. GANs are a very recent development in the field of deep learning that can learn to create data that doesn’t exist in the database^23,24^. Few researchers have attempted to generate microstructures directly using different variants of GANs^25–31^. Jangid et al. developed a GAN that could generate random grain shapes^26^ which were validated by comparing with real grains. CNNs on the other hand are kernel based neural networks that can learn various receptive kernels to be applied on the image data for classification and regression purposes. Here, CNNs are used as a post processing step to determine the size of generated synthetic defects.

The generated defects are placed in material space respecting the global distributions of defect features and also the spatial pattern. The uniqueness of the generated microstructures is maintained by applying a Poisson distribution over the mean number of defects while exploring different K-functions similar to that of real specimens.

## Methods

### Spatial point pattern

Spatial point pattern (SPP) analysis is a branch of study in stochastics mainly used in the field of astronomy, ecological survey etc. SPP is any point or location in a specified region (defects in material space in our case). These events occur randomly and can be modelled with a specific stochastic process. As discussed by^16^, SPP can be divided into three main categories:

- *Random or complete spatial randomness (CSR):* where the points or events are randomly distributed and can be modelled via Poisson process.
- *Clustered:* where points or events attract to each other in space forming small groups called clusters.
- *Regular:* where the points repel each other.

A point pattern analysis (PPA) is mainly concerned with describing and making sense of the process that could have generated these random patterns, for example: the occurrence of defects in a material is controlled by various parameters linked to material thermodynamical properties. A PPA can be described with two properties^32^, namely:

- *First order properties:* are the descriptions on the basis of intensity functions, density of defects at a particular material space for example.
- *Second order properties:* are the descriptions on the basis of interactions between each event or points in their material space.

First order properties are studied over a sub-region for a large number of events or points. Variations in these properties from each sub-region to another can make the point pattern inhomogeneous. The first order properties are helpful for a global spatial distribution analysis but aren’t efficient to distribute the defects spatially in material space. Moreover, global parameters like volume fraction of defects in a specimen has no strong relationship with fatigue life. Hence, patterns of defects in space are studied via second order properties which include: Nearest Neighbor function (NND), Ripley’s K-function, etc. Second order properties are those where an occurrence of each event is linked or dependent on one another and characterized by the distance between them. Point patterns are statistically compared with complete spatial randomness (CSR) of the null hypothesis for a thorough analysis.

### Poisson process

CSR is a state where events or points occur randomly in space with no interactions between each other. CSR forms the continuum of the natural ordering or patterns of events. On the either side of this continuum lies clustered and regular state of point patterns as explained by^33^. CSR can be modelled with just one parameter such as the expected density of points in space. This can be done via Poisson process since any random event follows a Poisson distribution with a mean value or expected value (density of defects in this case) which is given by,

1 $$\begin{aligned} P\{N(V)=k\}=\frac{(\lambda )^k}{k!}\exp ^{-\lambda } \end{aligned}$$

where $\lambda$ is number of points per unit volume, sometimes also called as rate parameter, *V* is the volume of material space in our case and *N* is the possible random variable. Equation 1 gives the probability of *N* being equal to *k*. For *n* disjoint sets $V_1, \dots , V_n$ the random variables $N(V_1), \dots , N(V_n)$ are independent of each other i.e., each point is stochastically independent and there exists no interaction between them which defines CSR. This stochastically independent state of point pattern is therefore often used as reference to evaluate if a point pattern is clustered or dispersed (attracting or repulsing).

### Univariate and bi-variate Ripley’s K-function

Ripley’s K-function is an effective tool to quantify second order properties of a spatial point pattern. With distance between each pair of events or points as the main parameter, Ripley’s k-function can estimate the probable number of events or points that can be found within a particular distance. K-function can be expressed in multiple variants. When all the points in the study region belong to one type or class, it is said to be univariate K-function and when the points are divided into two different types or classes, it can be called as bivariate K-function. In general form, K-function is given by,

2 $$\begin{aligned} K(d) = \lambda ^ {-1}E[\text {number of events within distance }d\text { of a randomly chosen event}] \end{aligned}$$

which is given as,

3 $$\begin{aligned} K(d) = \frac{V}{N} \sum _{i=1}^{N} \sum _{i\ne j}^{N} \frac{I(r_{ij}<d)}{N} \qquad I \rightarrow \left\{ \begin{array}{ll} 1, &{}\quad \text {if } r_{ij}<d \\ 0, &{}\quad \text {otherwise} \end{array} \right. \end{aligned}$$

where *V* is the volume and *N* is the total number of points in material space. *V*/*N* is nothing but $\lambda ^{-1}$ which is the intensity of events (points) or the number of points in a unit volume and *I* is the identity operator which equals to one if the distance between point *i* and *j* is less than distance *d* and equals to zero otherwise. The value of K-function is usually compared to the theoretical value of *K* for CSR or homogenous Poisson process. From the null hypothesis for CSR, Ripley’s K-function reduces to the volume of the sphere with radius equal to distance *d*,

4 $$\begin{aligned} K_{Poisson}(d)=\frac{4}{3}\pi d^3 \end{aligned}$$

The deviation of K from the theoretical value can estimate the nature of spatial distribution of events. If $K(d)>K_{Poisson}(d)$, the pattern is said to be clustered and vice versa.

In bi-variate K-function, the events or points are classed into two types, for example, Orange trees and apples trees, stars and planets etc. Bivariate functions as a whole can be represented in matrix form where $K_{11}$ and $K_{22}$ are K-functions of type 1 and type 2 points. The intensities of each type $\lambda _1$ and $\lambda _2$ are the two variables of the bi-variate K-functions. The interaction between these two processes is measured with cross K-function $K_{12}$. The procedure to measure $K_{12}$ remains the same as univariate K-function except that within a sphere or circle, number of other type points are counted. Cross K-function is given as,

5 $$\begin{aligned} K_{12}(d)=(\lambda _1 \lambda _2 V)^{-1} \sum _{i=1}^{N_1} \sum _{j=1}^{N_2} I(r_{ij}<d) \end{aligned}$$

where, $\lambda _1$ and $\lambda _2$ are the intensities of type 1 and type 2 points, $N_1$ and $N_2$ are the number of type 1 and type 2 points or events.

### Estimation of parameters for Neyman–Scott process

If a pattern is homogeneous, Poisson process can generate the pattern whilst for inhomogeneous patterns, strategies like Neyman–Scott process, Strauss process or Matern process needs to be used. The parameters for such processes needs to be estimated in prior^21^. In the current work, Neyman–Scott process is used where the points are classified into two types: parent and children. Parent forms the center around which children points are distributed with a known distribution whilst parent points are distributed homogeneously in space.

To classify the defects into types, a parameter $\theta$ was introduced in K-function. This parameter can be called as threshold size parameter with which defects are classified based on their sizes. With the introduction of $\theta$, *K* and $K_{12}$ functions can be expressed as,

6 $$\begin{aligned} K_{kk}(d,\theta )= & {} \frac{V}{N_k(\theta )}\sum _{i=1}^{N_k(\theta )} \sum _{i \ne j}^{N_k(\theta )} \frac{I(r_{ij}<d)}{N_k(\theta )} \end{aligned}$$

7 $$\begin{aligned} K_{12}(d,\theta )= & {} (\lambda _1 \lambda _2 V)^{-1} \sum _{i=1}^{N_1(\theta )} \sum _{j=1}^{N_2(\theta )} I(r_{ij}<d) \end{aligned}$$

where, $k = 1,2$ depending on type of defect, $\lambda _1 = \frac{N_1(\theta )}{V}$, $\lambda _2=\frac{N_2(\theta )}{V}$, $N_1(\theta )$ and $N_2(\theta )$ are the number of type 1 and type 2 defects.

### Data acquisition

Four reference samples of IN100 (labeled A, B, C and D, see Fig. 1) were characterized by X-ray tomography with a Nikon XT H 450 using a voxel size of 25 ${\upmu }$m. XCT images of these specimens were then processed and the defects were segmented to create binary masks. Each connected defect volumes were labelled separately such that they are identified and accessible in the segmented volumes. For the training of GAN and CNN, each defect volume was cropped out from XCT image and rigorous augmentation techniques were applied to increase the size of database via random rotation, flip etc. Finally around 1200 pores and 1200 shrinkages were resized to a shape of $32\times 32\times 32$ pixels for pores and $64\times 64\times 32$ pixels for shrinkages. Pixel values of the dataset were normalized from [0,255] to [0,1].

### Deep learning networks

Two Deep learning neural networks are integrated together in this work to generate synthetic defect: GAN and CNN. GANs are a generative model which usually contains two blocks of networks namely, generator and discriminator. The generator ${\mathcal {G}}$ takes in random 1D vector *z* and generates a 3D image of defect volumes while discriminator ${\mathcal {G}}$ is trained with both real ${\mathcal {D}}(x)$ and generated image ${\mathcal {G}}(x)$ to predict if the image is real or fake. The generator tries to minimize the value function of discriminator whilst the discriminator tries to maximize it. Hence, approach of GANs are sometimes also referred to as minmax game:

8 $$\begin{aligned} \underset{{\mathcal {G}}}{min}\>\underset{{\mathcal {D}}}{max}\>V({\mathcal {D}},{\mathcal {G}})={\mathbb {E}}_{x\sim p_{data}(x)}[log({\mathcal {D}}(x))]+{\mathbb {E}}_{z\sim p_{data}(z)}[log(1-{\mathcal {D}}({\mathcal {G}}(z)))] \end{aligned}$$

where $p_{data}$ is the distributions pertaining to real images and *z* is the input distributions to generator ${\mathcal {G}}$.The convergence of the network is reached when the generator successfully fools the discriminator and discriminator fails to predict authenticity of the image. Theoretically, the value function at convergence is 0.5. In the current work, a DCGAN inspired architecture has been used with binary cross entropy loss function. CNNs on the other hand are fairly simple to train. The network is trained to predict the actual width, height and depth of the real defects by training on the resized images^34–36^. The convergence is achieved by minimizing the mean squared error between the actual and predicted size via stochastic gradient descent.

#### Network structure

In our model, generator takes in a normally distributed random input vector of size 128. The input layer is connected to a fully dense layer followed by 3 transposed convolutional layers and a convolution layer with kernel size of 4 and a stride of 2. Batch normalization and ReLU activation layers are added in between except in the last convolution layer and finally a sigmoid layer at the end. Discriminator on the other hand is an exact mirror of generator except for the last layer which is one single output. Furthermore, the ReLU layers are replaced with Leaky ReLU activate layers. A gaussian kernel initializer is used to assign initial values of weights and bias with a mean of 0 and standard deviation of 1.

The architecture of CNN contains 4 convolutional layers along with max pooling layers of size 2. ReLU activation layers are added between each convolutional and max pooling layers followed by a dense fully connected layer and 3 linear output neurons at the end.

#### Training procedure

For the GAN, a batch size of 16 was used with adaptive moment estimation optimizer (ADAM)^37^. Learning rate for generator was set 2 times that of discriminator with a value of 0.0002. Generally in the Vanilla GAN, the generator is updated once per each update of discriminator. As a result of which the discriminator learns quicker when compared to generator. Therefore the generator is trained twice for each update of the discriminator to keep the balance between the training of generator and discriminator. Furthermore, hyperparameters such as learning rate of discriminator and generator, decay parameter of optimizer, number of filters of each layer etc. were tuned via a random search method. Model performance was seen to largely depend on learning rates and the number of filters associated to each layer. Initially, vanishing gradient problems were encountered during the training. However, adding batch normalization layers along with one sided noise smoothing of labels fixed the issue. One sided label smoothing is a method to add a small noise to the labels of discriminator^38^. A random noise of ± 2% was added to labels.

CNNs also use ADAM optimizer with a learning rate of 0.001 and batch size of 32. CNN is converged by minimizing the mean squared error.

## Results

### Spatial point pattern

**Figure 2:**
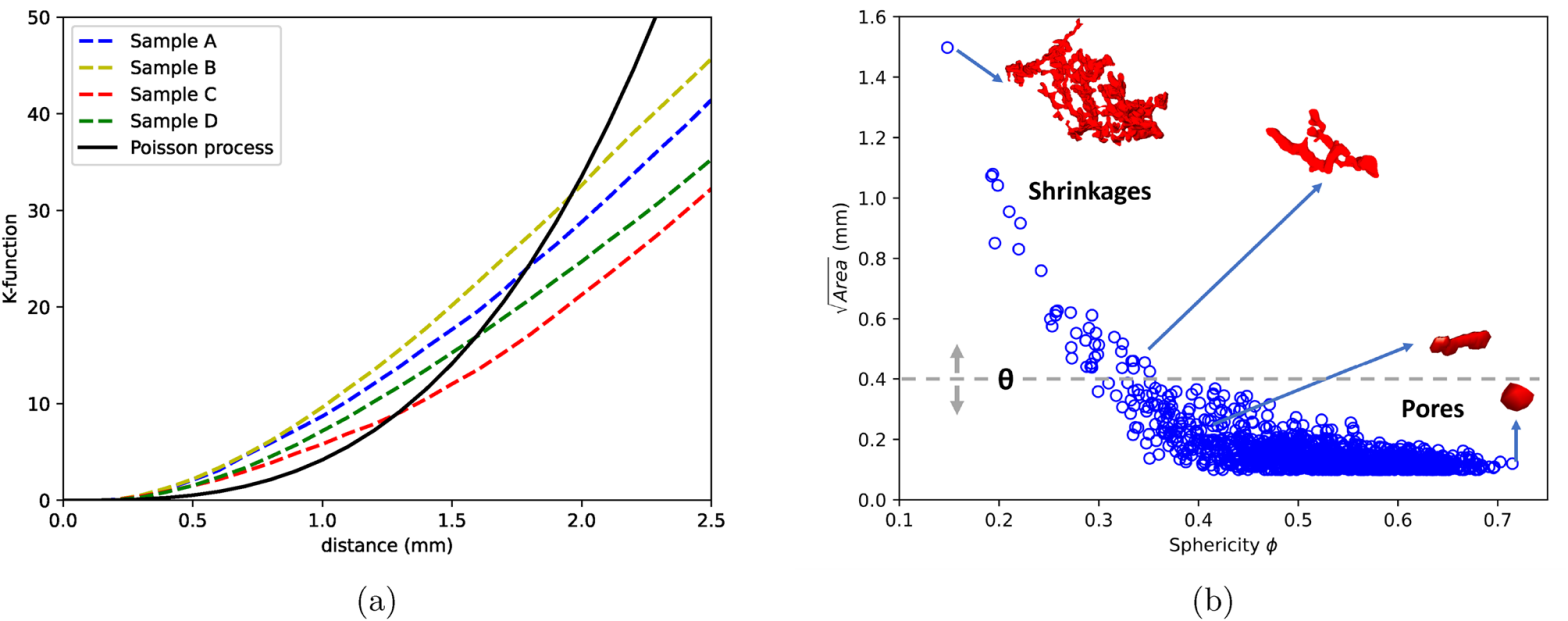
(**a**) K-functions of samples comprising all defects showing the aggregation and dispersion effects (see text for more details). (**b**) Defect size vs sphericity displaying the evolution of morphology along the defect size with shrinkages being very tortuous while pores more spherical in shape.

Spatial point patterns of defect distribution in 4 reference microstructures of IN100 were analysed via Ripley’s K-function^14,21^. K-functions are compared to the theoretical value of K for complete spatial randomness (CSR) or homogeneous Poisson process^39,40^. Any deviations from Poisson process indicates the nature of spatial pattern i.e., if $K(d)>K_{Poisson}(d)$, the points are said to be attracting or clustered and vice versa. From Fig. 2a, strong clustering effects are seen in short distance ranges ($K(d)>K_{Poisson}(d)$) and dispersion in large ranges ($K(d)<K_{Poisson}(d)$). To simulate such an inhomogeneous pattern, different strategies like Strauss process, Matern process or Neyman–Scott process need to be employed^21^.

The defect size is plotted as a function of sphericity in Fig. 2b. Defect size is defined by $\sqrt{Area}$ where *Area* is the projected area of defect on a plane perpendicular to loading direction^6^ while sphericity is a morphological parameter which measures how spherical a defect is: a value of 1 indicates a perfectly spherical defect^15^. Sphericity, $\phi$ is given by $\frac{\pi ^{1/3} V_p}{A_p}$ where $V_p$ is the volume of defect and $A_p$ is the surface area. Figure 2b shows a clear inverse relationship between defect size and sphericity (defects get more and more spherical as their size reduces). Indeed, small pores are mostly formed due to trapped gases while larger pores are shrinkages and tend to be much more tortuous as the size increases. From Fig. 2b, it can be seen that defect size ranges from 100 µm to 1.5 mm. Due to this large variance in defect size, the K-function was modified to assess attraction or repulsion among specific groups of defects (classified based on their size). A defect size threshold $\theta$ which is a $\sqrt{Area}$ value was introduced to classify defects into two groups (see section “Estimation of parameters for Neyman–Scott process”), the two groups being shrinkages (larger defects) and pores (smaller defects). By varying $\theta$, it is possible to investigate the existence of two different processes in the formation of voids via bivariate K-functions (see section “Univariate and bi-variate Ripley’s K-function”). By splitting the defect into two groups as type 1 for defects of size larger than $\theta$ and type 2 for defects smaller than $\theta$, it is assumed that defects of type 1 and type 2 are two different processes for which K-functions and cross K-functions are analysed. Defects are initially classified at $\theta =$ 1 mm and varied upto $\theta =$ 0.1 mm.

Cross K-function is a method to estimate interaction between two processes i.e., the spatial ordering of type 2 defects around type 1 defects^21^. This kind of analysis helps to understand if the smaller defects are aggregated with respect to each other or with the larger defects and furthermore aids to simplify the simulation of in-homogeneous point process. Bivariate K-functions together can be described in the form of a symmetrical matrix given that pattern is stationary where, $K_{11}$ and $K_{22}$ are K-functions of type 1 and type 2 defects and $K_{12}$ is the cross K-function between point process of both type of defects. In other words, $K_{11}$ is K-function of all defects larger than $\theta$ and $K_{22}$ for the defects smaller than $\theta$. As $\theta$ reduces, defects from type 2 group are moved to type 1 group.

**Figure 3:**
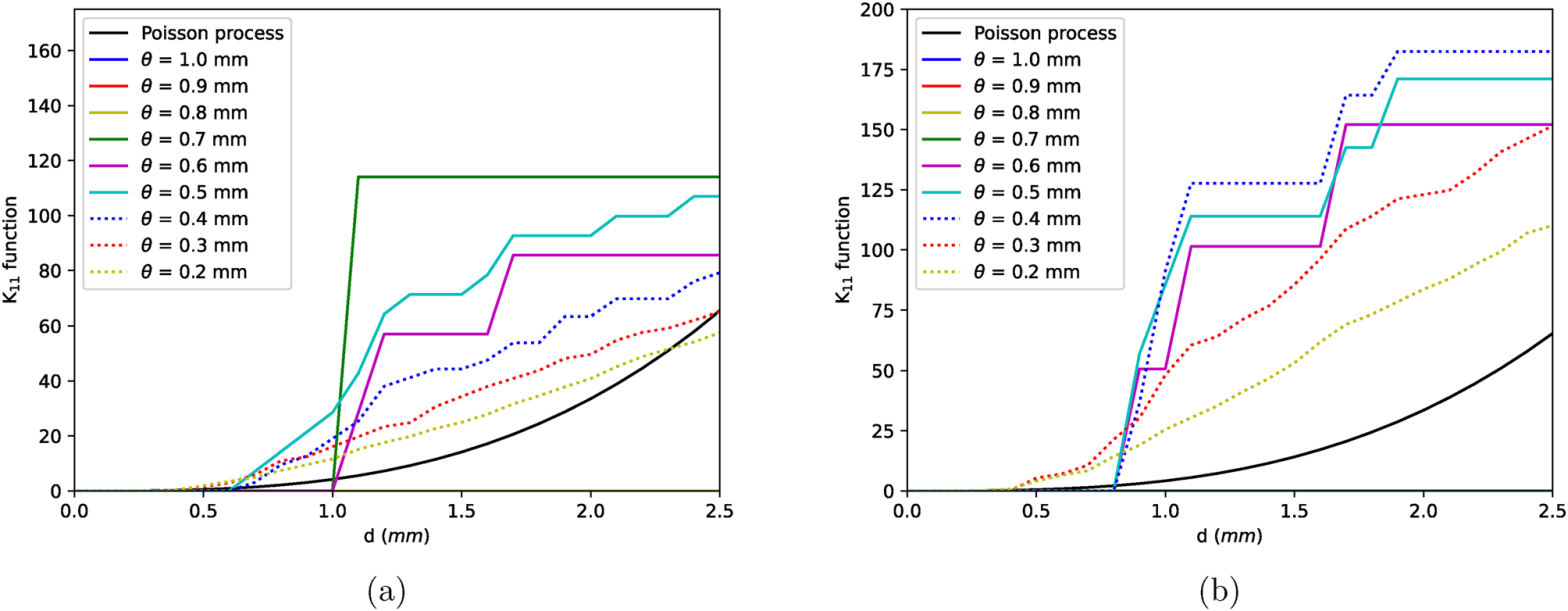
$K_{11}$ functions for (**a**) sample D, (**b**) sample B for different values of $\theta$.

**Figure 4:**
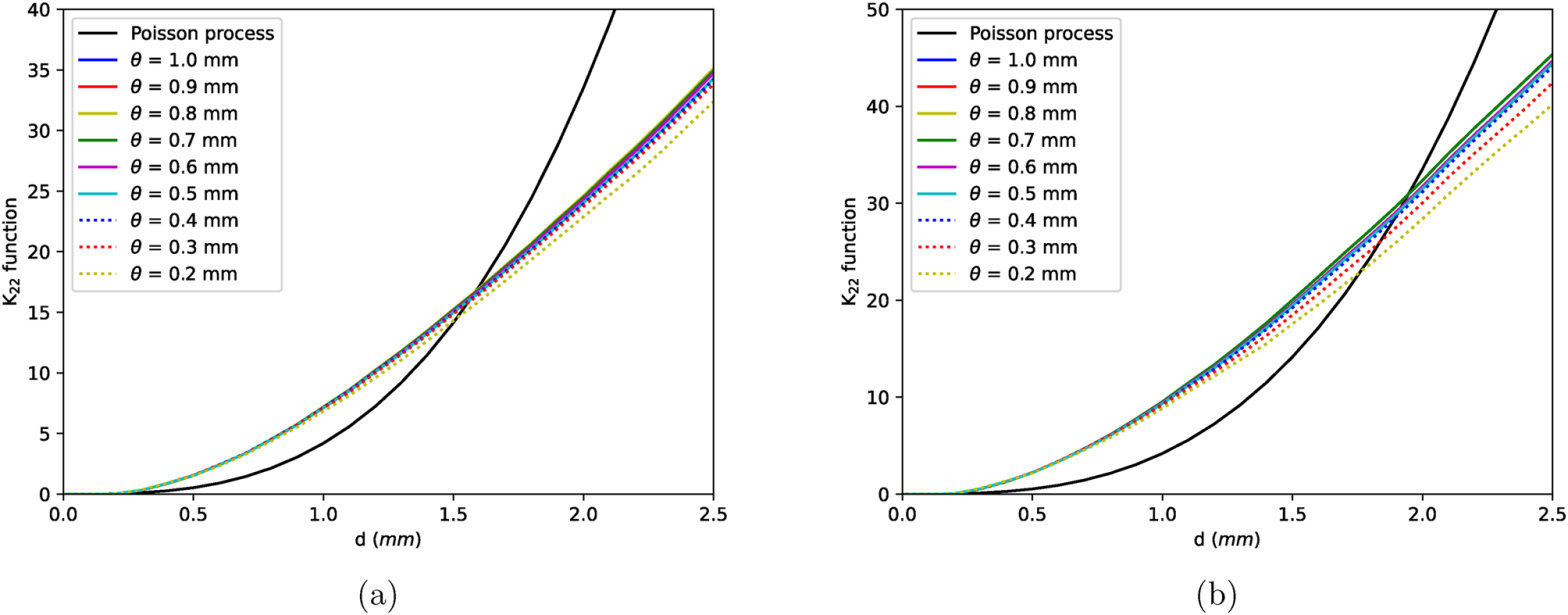
$K_{22}$ functions for (**a**) sample D, (**b**) sample B for different values of $\theta$.

**Figure 5:**
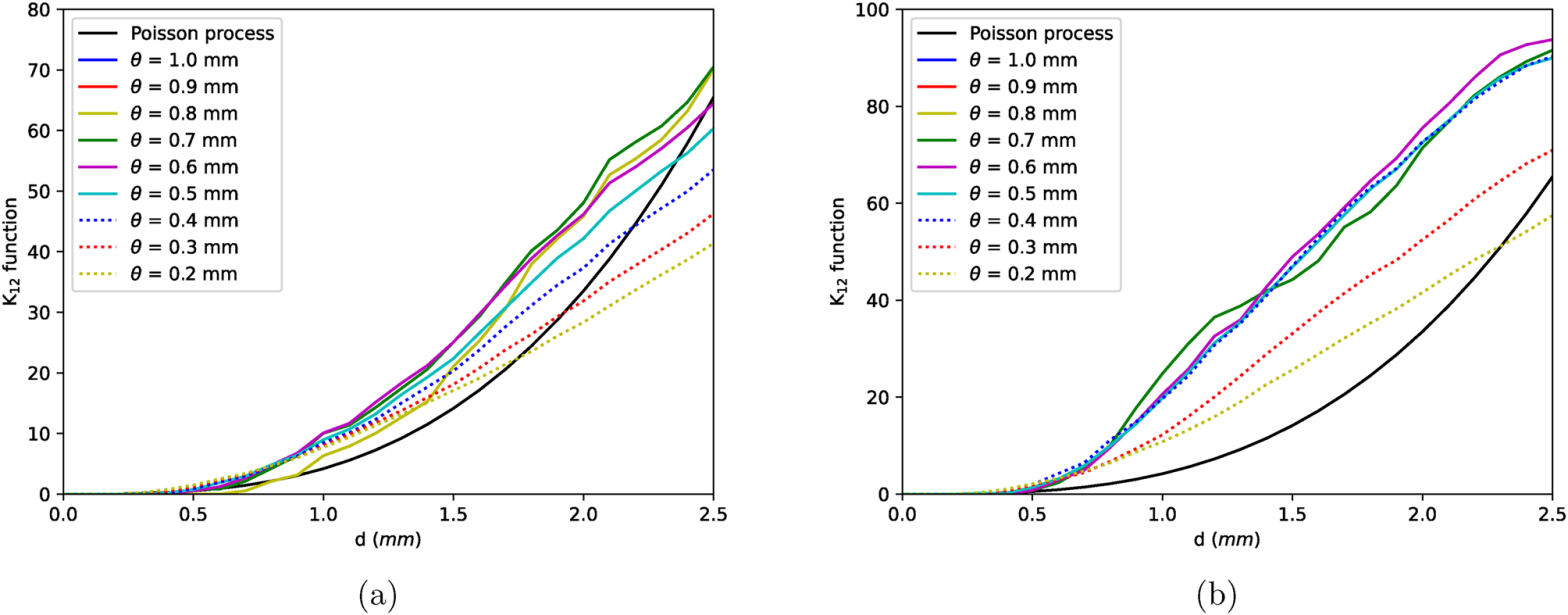
$K_{12}$ functions for (**a**) sample D, (**b**) sample B for different values of $\theta$.

Results from $K_{11}$, $K_{22}$ and $K_{12}$ functions of two reference samples are shown in Figs. 3, 4 and 5. From Fig. 3, strong aggregation can be seen among defects larger than approximately 0.4 mm ($\theta$ values above 0.4) but as the smaller defects are considered, the clustering effect reduces (with respect to $\theta$). This reduction is due to the fact that smaller defects which are necessarily pores are spread across the length of the sample. Similar effect can be seen for $K_{22}$ functions where the function remains more or less same for $\theta$ values between 1–0.4 mm and reduces thereafter signifying that clustering is driven by defects larger than 0.4 mm.

The interaction between the two classes of defects with respect to parameter $\theta$ can be described with cross K-function $K_{12}$. At a given $\theta$, $K_{12}$ function measures if the defects smaller than $\theta$ are clustered or dispersed with larger defects. From Fig. 5b, it is seen that smaller defects are strongly clustered with larger defects upto a value of 0.4 mm. However, in some samples $K_{12}$ function reduces marginally with respect to $\theta$ even for values greater than 0.4 mm. This reduction is attributed to the existence of secondary and tertiary clusters apart from one large primary cluster as depicted in Fig. 1d. The weak attraction of these subordinate clusters which contains defects larger than 0.4 mm reduces the cross K-function as seen in Fig. 5a. Similar effect can also be seen in $K_{11}$ function of this sample, see Fig. 3a.

Given the fact that in most scenarios K-functions remains nearly constant upto a $\theta$ of 0.4 mm, it appears that defects above and below this size follow different processes of void formation mechanisms. One of the processes is where the smaller defects are nucleated randomly across the length of the sample while the other where the voids are localised to form clusters i.e., the larger defects whose K-functions shows strong aggregation. These two processes interact with each other causing the smaller defects to be attracted towards larger defects. This can also be seen in Fig. 1d where defects smaller than 0.4 mm (colored in blue) are spread across the sample but interacts with larger defects (colored in red) to form clusters. Those larger than 0.4 mm are certainly shrinkages which are tortuous in shape as seen in Fig. 2b whose formation is linked to thermodynamical processes of solidification whilst the rest are pores formed mostly due to trapped gases. Nevertheless, from Fig. 4 negligible attraction effect is seen even for pores ($K_{22} > K_{Poisson}$) due to the interaction between two processes. It is however important to note that the clustering effects at all $\theta < 0.4$ mm for $K_{11}$ and $K_{12}$ functions are not caused by the same effect. In these functions, defects larger that $\theta$ are included in the calculations i.e., for example, at a $\theta$ of 0.1 mm, $K_{11}$ function is measured for all defects larger than 0.1 mm. Therefore, in these functions the clustering effect for lower $\theta$ values is induced by the larger defects. Finally, with the knowledge of existence of two processes and the interaction between them as described by bivariate K-functions, Neyman–Scott process can be used to generate such an in-homogenous point pattern. In this process, the parent events or defects are distributed homogeneously in the material space and children defects are distributed around the parent defects^41^. Shrinkages or defects larger than 0.4 mm typically found in the defect cluster are the parent defects whilst the pores are children defects.

**Figure 6:**
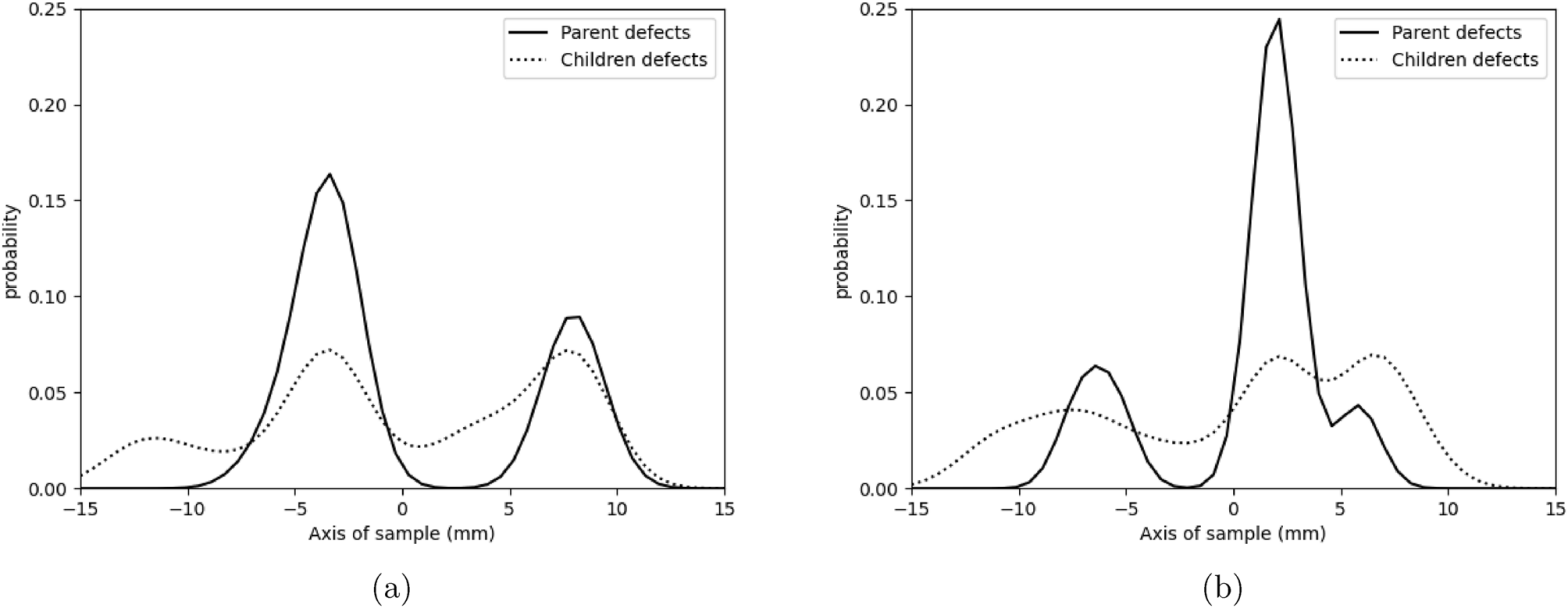
Distribution of defects along the length of sample (**a**) D, (**b**) C showing the existence of multiple clusters.

However, nucleation of parent defects is in-homogeneous and occurs at specific points along the length of the sample defined by a mixed Gaussian distributions as seen in Fig. 6. Furthermore, it is seen that children defects follow the same distribution along the axis of the sample due to the interaction between the two processes as already invoked. More importantly, the presence of multiple clusters (see Fig. 1) is accounted for by the number of gaussians of this mixed gaussian distribution.. Mixed Gaussian distributions or Gaussian mixture models (GMM) are characterised by means $\mu _{k}$, standard deviation $\sigma _{k}$ and weights $\pi _{k}$ where *k* is the number of Gaussians^42,43^. Via expectation maximization algorithm, respective means, standard deviations and weights of each Gaussians can be found. Average standard deviation of the parent defects’ Gaussians was approximately 9 pixels or 225 $\mu m$ whilst the means were found to be coherent with those of children defects as also seen in Fig. 6. Each Gaussian of the parent defects acts as a seed for the nucleation of clustered defects in that zone of the sample. This preference of clustering along the length of the sample may be due to solidification processes of cylindrical ingot bars which are used to machine the samples. Furthermore, it can also be due to the choice of location and orientation of samples to be machined from ingot bars: the axis of samples were placed parallel to the axis of the ingot bars during machining.

### Generation of synthetic defects

Morphology of the defects varies with respect to its size in an exponential pattern^15,44^. It is difficult to train GANs to reproduce defects that can respect this relationship since all defects will be initially resized to a fixed size for training. Hence GANs were discretized into two parts i.e., two adversarial networks were trained to generate defects: one for shrinkages (defects > 0.4 mm) and the other for pores (defects$< \theta =$0.4 mm). $\theta$ here is the threshold parameter as determined via SPP analysis. Since the number of shrinkages and pores were insufficient to train the network, a rigorous data augmentation step was carried out to increase the database size. The individual defect volumes were randomly rotated in 3D with angle bounds of $-45\deg$ to $+45\deg$, flipped and inverted in the data augmentation step. All defects were then resized to a fixed size of $64\times 64\times 32$ voxels for shrinkages and $32\times 32\times 32$ voxels for the pores before training the adversarial networks. The resizing of images is done by applying a zero order interpolation function $Image_{resized}=D(Image)$ where *D* is the interpolation function. To maintain the balance between generator and discriminator networks, the generator is updated twice per each update of discriminator. Furthermore, adding a small noise to the labels of discriminator has shown to improve the training of the adversarial network. The adversarial and discriminator loss balance out after as less as 5 epochs and the model would be trained within 60 epochs.

**Figure 7:**
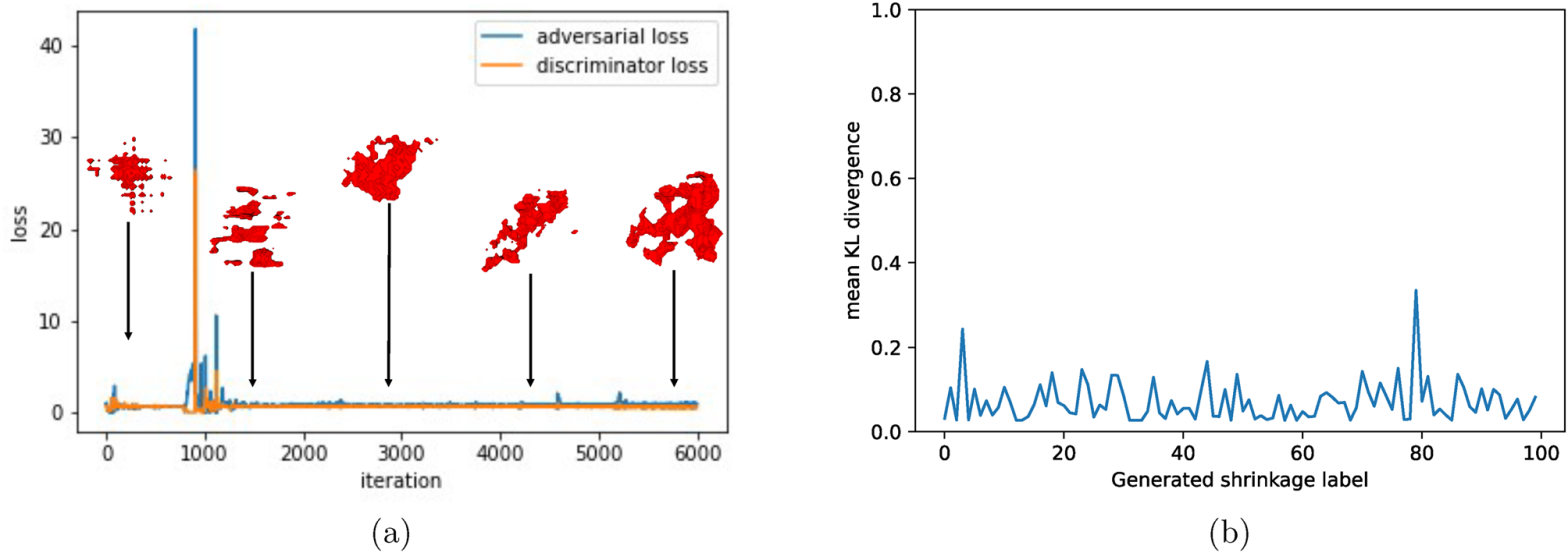
(**a**) Evolution of loss of GAN and generated defect along the training period with iterations being each update of discriminator. (**b**) KL divergence value between gaussian curvature of generated shrinkage and mean gaussian curvature of real shrinkage showing that generated shrinkages are similar to real defects.

**Figure 8:**
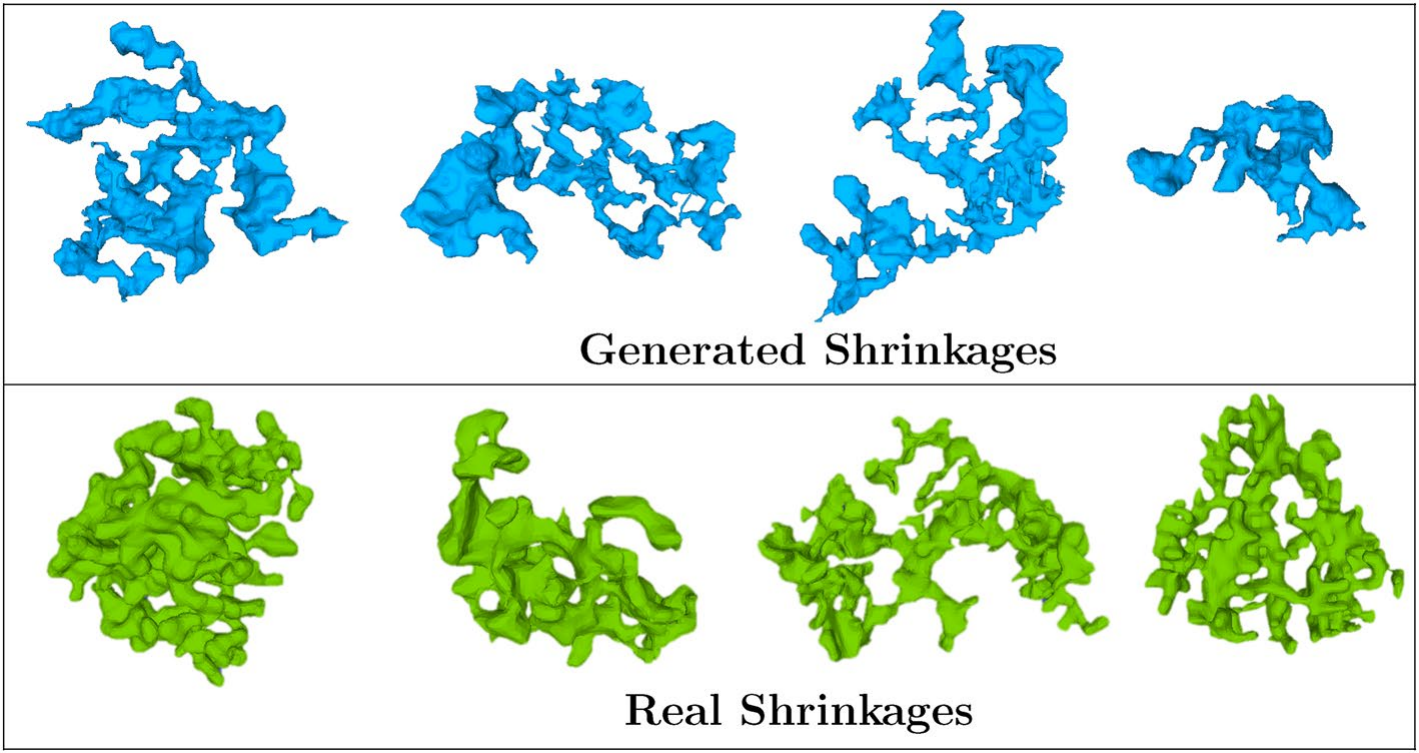
Examples of few generated shrinkages along with real shrinkages.

Since the generated defects are also of a fixed size similar to training data, CNNs are used to learn the inverse of the interpolation function used and to find the dimensions of defect’s 3D slices. Since a relationship is assumed between defect size and morphology, it is fairly an easy and quick process to train the CNNs^34,35^. The trained generators and CNNs can then be integrated to generate defects of various sizes and morphologies for the synthetic microstructures. Trained generator generates the defect and subsequently the trained CNN predicts its original size. The defect (3D image stack) is then upsampled and filtered to remove disconnected volumes as the final procedure in the generation of defects i.e. the largest volume is retained^45^.

**Figure 9:**
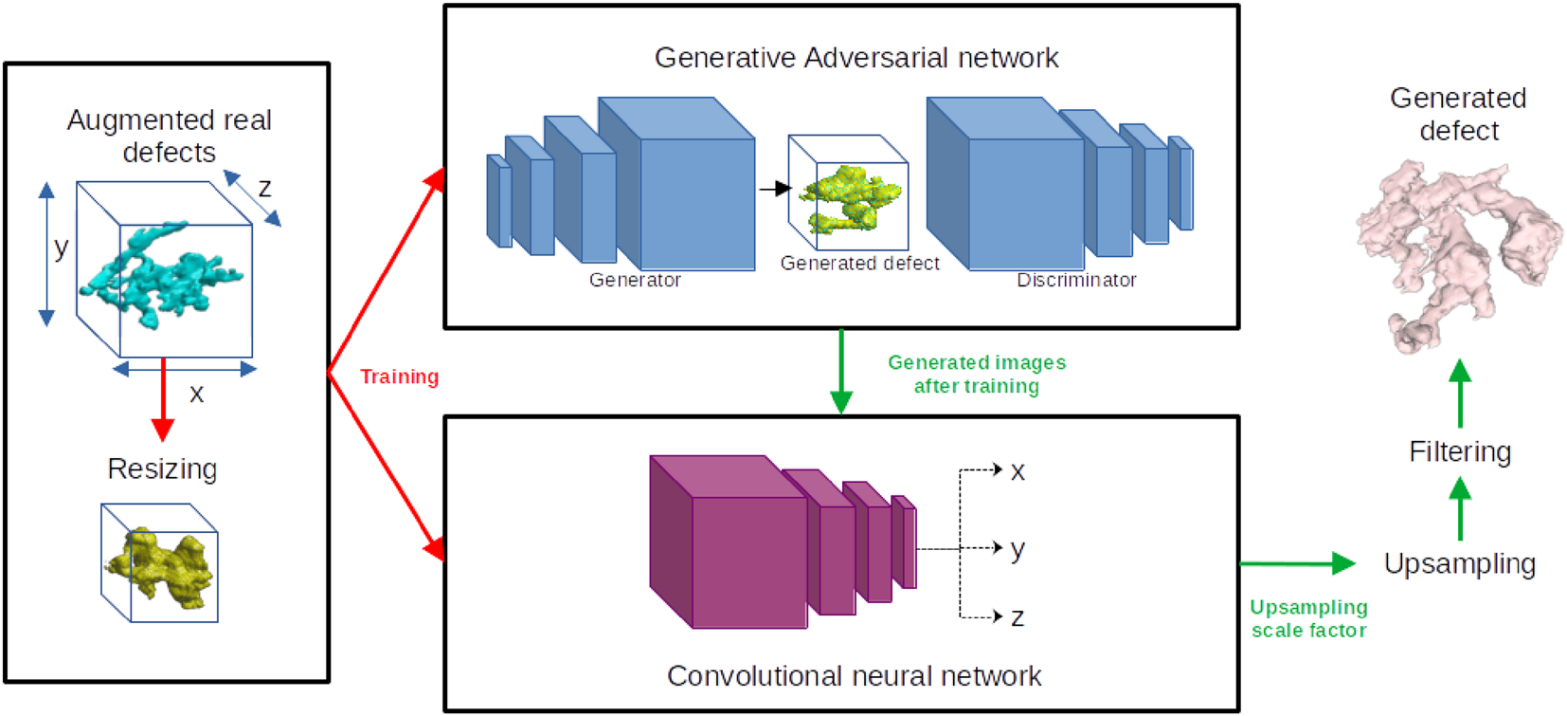
Representation of procedures for the generation of synthetic microstructures.

Evolution of the adversarial and discriminator loss of GAN is shown along with the evolution of generated defects with each discriminator update in Fig. 7a. The generated defects are validated by comparing local Gaussian curvatures^46^ of generated and real defects. Gaussian curvatures are defined as product of principal curvatures (or eigen vectors of local curvatures) at each vertex of the surface mesh. In this work, the gaussian curvatures are measured as per the methods described by^47^ using the python module trimesh^48^. Gaussian curvatures measured at all points on a given defect’s surface forms a gaussian distribution. For each generated defect, the distance between its gaussian curvature distributions with the mean distribution of real defects is measured via Kullback–Leibler (KL) divergence distance^49^ which is given by, $\log \frac{\sigma _R}{\sigma _G} + \frac{\sigma _G^2 + (\mu _G - \mu _R)^2}{2\sigma _R^2} - \frac{1}{2}$ where $\sigma _G$ is the standard deviation of gaussian distribution of generated defect, $\sigma _R$ average standard deviation for real defects and $\mu _G$ and $\mu _R$ are the respective means. Smaller the value of KL distance, more similar the two distributions are while for identical distributions, the value equals 0. As shown in Fig. 7b, this distance metric remains low exhibiting similarity with real defects and at the same time, each generated defect is unique as seen in Figs. 7b and 8. The entire procedure of synthetic defect generation via GAN and CNN is summarized in Fig. 9.

### Synthetic microstructures

The generation of a synthetic microstructure starts with the number of defects in the microstructure defined by drawing a Poisson random number with $\lambda = E(N_{real})$ where $N_{real}$ is the number of defects in real specimens. Through statistical estimations (ln-likelihood), generalised extreme value (GEV) was seen to best fit the defect size distribution in this material. Therefore, identified parameters of GEV distribution were used to estimate and generate number of defects for given size ranges discretely via our combined GAN and CNN model that produces unique synthetic defects as explained in previous section. These generated defects are placed in material space using positions defined by K-functions. The distribution of defects in real specimens is heterogeneous, therefore a Neyman–Scott process is adopted to replicate this heterogeneity^41^ via bivariate K-functions. The shrinkages (defects > 0.4 mm) act as parent defects and pores (defects < 0.4 mm) as children events. Contrary to traditional method, a mixed Gaussian distribution defined on the axis of the sample is used to distribute shrinkages (parent defects) in material space. A mixed gaussian distribution is then applied, the number of clusters *k* being randomly selected from a normal distribution with mean $\mu _{k}$ and variance $\sigma _{k}$ computed from the reference samples whilst the weights $\pi _{k}$ are randomly attributed to each Gaussian distribution such that their sum equals unity. Each Gaussian of this GMM acts as seeds for the nucleation of primary and subordinate clusters. Shrinkages are placed in the material where their planar co-ordinates (radial positions) are randomly chosen whilst their position along the axis is extracted randomly from the mixed Gaussian distribution. The process generates a random $K_{11}$ function similar to those of reference samples.

Furthermore, the children defects (pores) are added around the parent defects (shrinkages) conserving the interaction between the two processes via $K_{12}$ function and interaction amongst the pores via $K_{22}$ functions. Since volume of material space is constant and the number of defects are defined by Poisson random number, expected number of defects within any given distance *d* can be computed using equations 6 and 7 with respect to maximum and minimum K-functions of reference sample. It is ensured that $K_{12}$ function of generated sample is always in between the lowest and the highest value of reference samples. To not over-constrain the addition of generated defects as per K-functions in the material space, a small tolerance value is added to bivariate K-functions such that K-functions of each generated microstructure is similar to real specimens but unique. This way, the entire generation process is randomized and each generated microstructure is a Poisson random output with $\lambda =$ characteristics of real specimens. During this process, attention is given to avoid overlapping of defects within themselves and with the material boundary.

**Figure 10:**
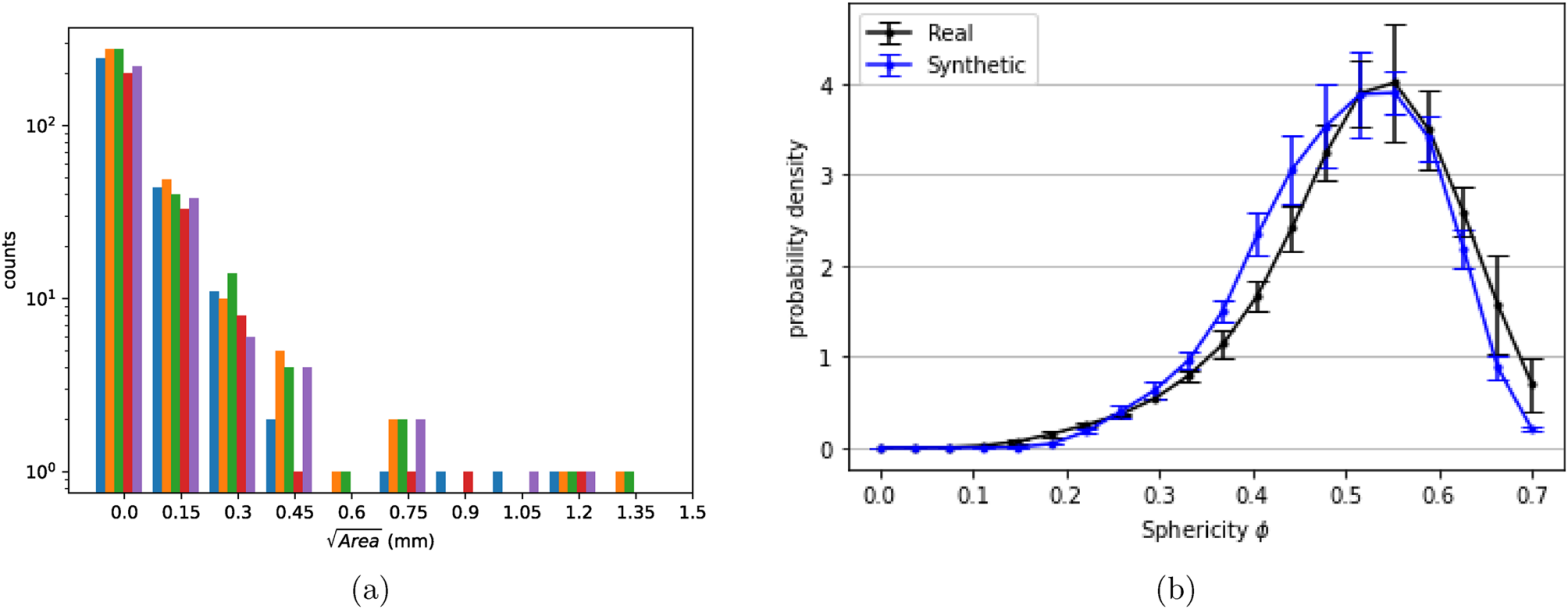
(**a**) Defect size distribution of 5 generated samples showing that each generated microstructure is unique in terms of total number of defects, parent and children defects and maximum defect size. (**b**) comparison of probability densities of Sphericity displaying the morphological consistency of generated microstructures with real microstructures.

Figure 10a shows the defect size distribution of 5 synthetic microstructures. Total number of defects of each synthetic microstructure is different since it is assumed that total number of defects follows Poisson distribution^50^. In other words, the number of defects, number of parent and children defects of synthetic microstructures are determined by the Poisson random number with metrics of reference specimens as rate parameter $\lambda$ i.e., average number of defects in samples.

Furthermore, sphericity of defects in the synthetic microstructures are compared in Fig. 10, the error bars represent 95 percent variances. Variance bars of generated microstructure’s sphericity distribution lies within the distribution of reference samples at almost all instants describing the morphological consistency of generated samples.

**Figure 11:**
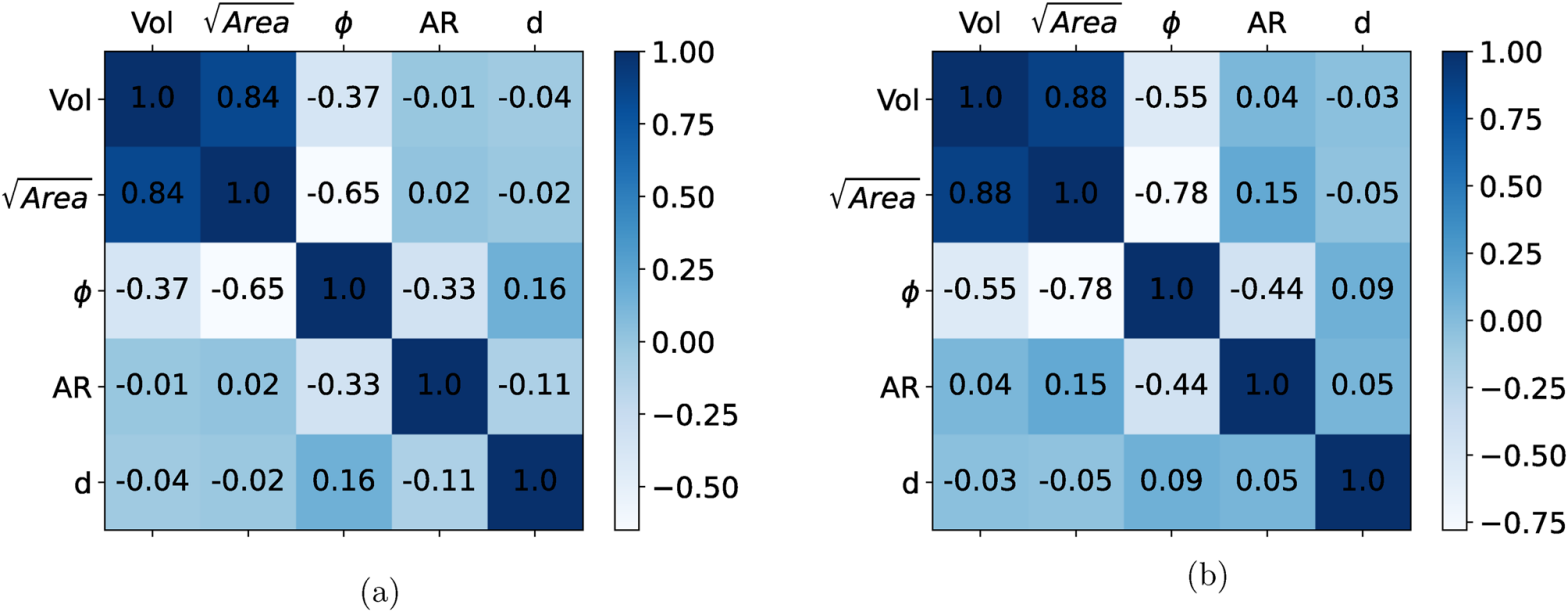
Comparison of global inter relationships between defect features via PCC (**a**) Real microstructure, (**b**) Synthetic microstructure, displaying the statistical coherency between synthetic and real microstructure in terms of inter-relationships between the defect characteristics.

**Figure 12:**
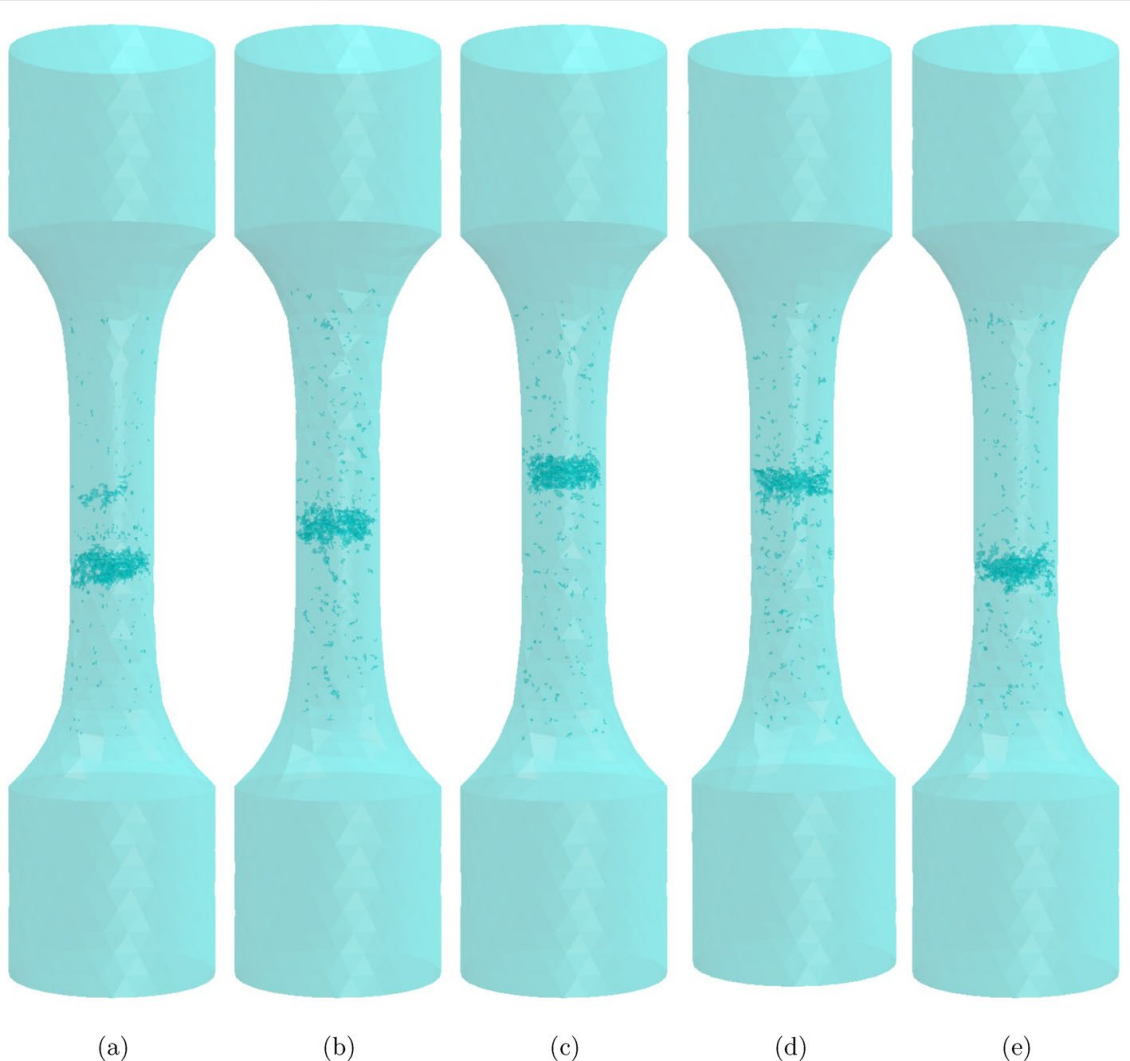
Examples of few generated samples.

Some of the morphological features are usually correlated in real specimens for e.g., $\sqrt{Area}$ and $\phi$ are negatively correlated^8^ as also seen in Fig. 2b. Defect size on the other hand, can be expressed in various forms like cube root of volume, equivalent radius of a sphere assuming volume of defect is equal to that of this sphere etc. However, $\sqrt{Area}$ is the one most used to describe fatigue as it allows to capture mode I crack propagation and is empirically linked to fatigue life, stress intensity factors of crack etc.^7–9,51–53^. Inter-dependencies of these features are found to have a prominence in fatigue performance of a material. The correlations between each of such features can be measured via Pearson Correlation Coefficient (PCC)^54–57^. Apart from these features, defect characteristics such as aspect ratio (AR) and distance from free surface (*d*) plays an important role in fatigue performance of the material too. AR is a ratio of major axis to minor axis of a defect projected on a particular plane (here, a plane perpendicular to loading axis which is the axis of specimen). Figure 11 shows the PCC between each of such features in the form of a matrix. It is seen that the generated microstructure preserves the inter-relationships between prominent defect features, given the similarities between PCCs of real and synthetic samples. Some of the generated synthetic microstructures are shown in Fig. 12.

With all these global statistics being in good agreement with those of real specimens, it can be said that the method applied for the generation is efficient and can replicate the real specimens in terms of spatial arrangement as well as the morphological and statistical aspects of defects. Furthermore, the uniqueness of each generated microstructure is conserved by the random number of defects that is defined by Poisson distribution, randomly explored K-functions along with new unique defects generated by Deep Neural Networks (DNNs) for each microstructures.

## Discussion

**Figure 13:**
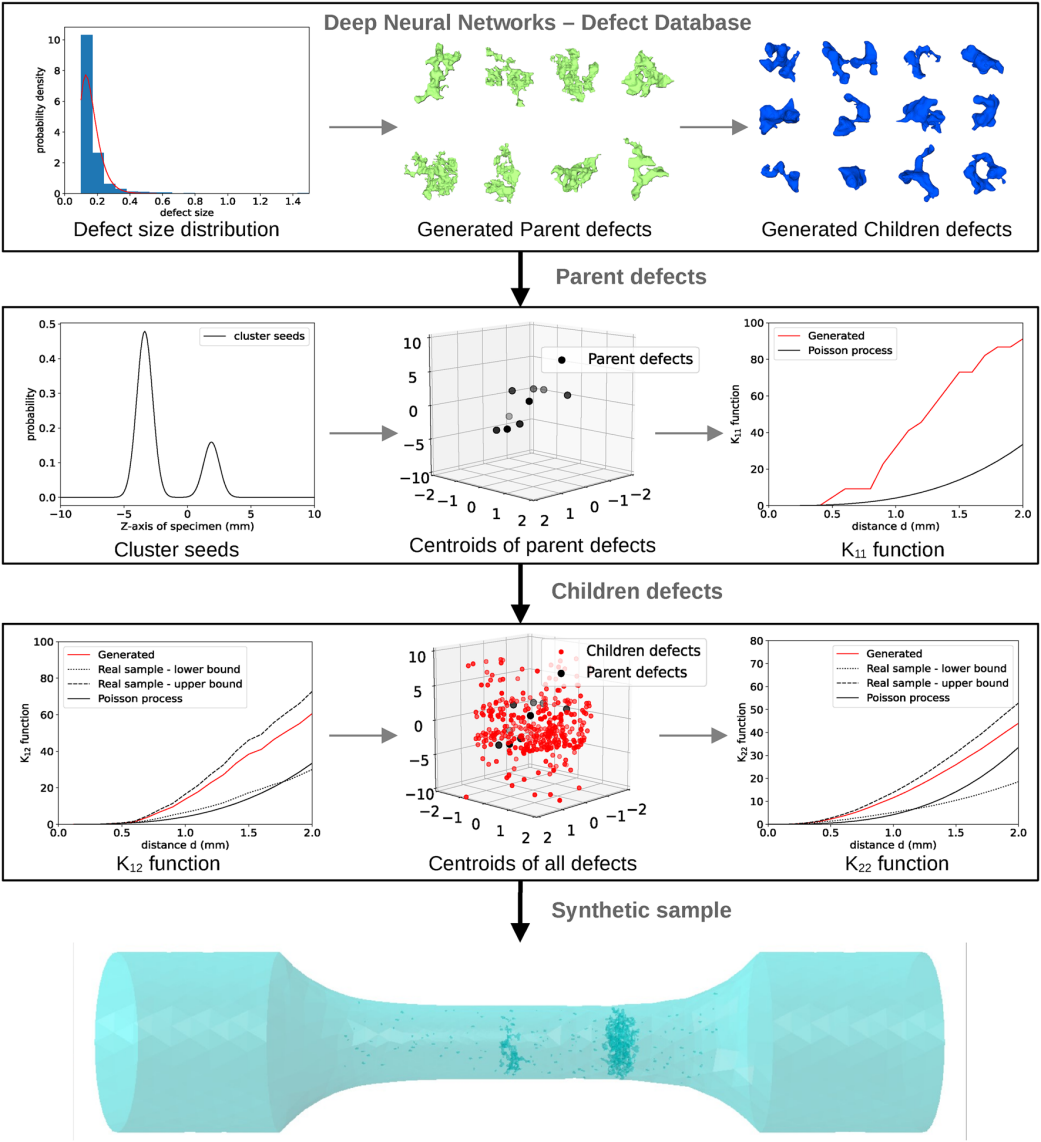
Illustration of the strategy to generate synthetic microstructures.

A novel strategy has been developed in this work to generate synthetic microstructure in a more cost-friendly and efficient way by integrating SPP analysis and GAN as illustrated in Fig. 13. Generating greyscale XCT-like images directly would have been impractical and very computationally demanding given the size of samples^58–61^. In addition, it would not have brought any additional information as ultimately the images would have been thresholded to segment the defects. Also, training such a model would require an enormous number of XCT images as input. In this regard, combined use of SPP and DNNs appears very effective.

Bi-variate Ripley’s K-functions were used to analyse SPP of real reference microstructures and to generate synthetic ones. Ripley’s K-function is usually affected by edge effects where the measurement domain of distance *d* goes out of the study-region. One of the simplest methods to avoid this error is to measure K-function only upto $1/3^{rd}$ of the largest possible distance. Hence, K-functions were analysed only until a distance of 2 mm in the above cases. Moreover, mechanical interaction of a pore with any shrinkage that is farther away than 2 mm from itself is nearly negligible given a gauge section diameter of 3.7 mm and maximum possible defect size of approximately 1.5 mm. Therefore, for all pores beyond 2 mm from any of the parent defects, a Poisson process was assumed to distribute them in material space.

Furthermore, to assess if the process behind the point pattern is different for larger and smaller defects, a defect size threshold parameter $\theta$ (which is $\sqrt{Area}$ value) was introduced to classify defects based on their sizes. K-functions were analysed at different $\theta$ values ranging from 1 to 0.1 mm. From our previous work^15^, defect population was classified into three groups: a) Shrinkages, b) broken shrinkage pores and c) gaseous pores. Statistically, all defects larger than approximately 0.3–0.4 mm are shrinkages due to their tortuous morphology as seen in Fig. 2b and matches with the findings of SPP analysis where a $\theta$ of 0.4 mm explicitly classifies the void nucleation mechanism into two groups: Shrinkages and pores. The clustering of defects is driven by shrinkages which nucleates at specific zones in material space and the gaseous porosity interacts with this process as shown by cross K-functions.

The shrinkages of reference samples were resized to a cuboid size of $64\times 64\times 32 (px^3)$ to train the generator given the unsymmetrical size of shrinkages in three directions. The average radial width of shrinkages (X and Y direction) was found to be around 50 px while the thickness along the axis (Z direction) was found to be 29 pixels. This difference might be linked to gradient of cooling rate along the radial axis of the ingot bars^62–64^. Furthermore, the current approach to generate defects can be further developed by various means given the increasing popularity of deep learning techniques in materials science^65,66^. For example, GAN can be conditioned to generate a defect of particular characteristics which would reduce the generation time for synthetic microstructure and give further control to the user^67^. Furthermore, a Deep convolution GAN inspired architecture was used in the current work which can be replaced with more advanced GAN networks such as Wasserstein GANs, Style GAN, Spatial GANs etc.^28,68,69^. It might also be possible to integrate the generation of grains into this existing model to also capture the effects of grains, slip plane etc. on material’s performance. In the current approach, CNN’s predict the X, Y and Z dimension of the generated defect which is later upscaled by interpolation. This can be replaced by bottle-neck architectures like U-nets to directly upscale the defect which should probably remove the filtering step in the model.

Assuming a similarity in granular characteristics in all samples, grains were not considered in the current approach. However, the approach is also compatible if such granular microstructure needs to be taken into account assuming that there is no correlation between grain size and defects. This can be easily done by generating grains by Voronoi tessellation as demonstrated by Quey et al.^70^ and performing a Boolean operation between the granular microstructure and microstructure containing defects for example.

With regards to further usage of this strategy, one immediate application would be to analyse the influence of each feature of defect on the number of cycles to failure in fatigue loading via fracture mechanics. Particularly in the case of clustered defects, such an analysis should aid in finding an approximate function that can better predict the fatigue life of samples taking into account inter-defect interactions. Furthermore, similar to approach of El Khoukhi et al.^71^, a Monte-Carlo like approach can be implemented to estimate fatigue life in a probabilistic fashion. The results of all these extended works with the aid of synthetic microstructure will be presented in our future articles.

## Data Availability

X-ray Tomographic datasets of reference samples as well as the codes (machine learning and synthetic microstructure generation module) are available upon reasonable request from the corresponding author.
